# Correlation study of serum lipid levels and lipid metabolism-related genes in cervical cancer

**DOI:** 10.3389/fonc.2024.1384778

**Published:** 2024-05-08

**Authors:** Lin Cheng, Zhuo Li, Qingmei Zheng, Qin Yao

**Affiliations:** ^1^ Department of Obstetrics and Gynecology, The Affiliated Hospital of Qingdao University, Qingdao University, Qingdao, China; ^2^ Department of Pediatric Surgery, The Affiliated Hospital of Qingdao University, Qingdao University, Qingdao, China

**Keywords:** serum lipid profile, lipid metabolism, prognostic model, genes, cervical cancer

## Abstract

**Objective:**

Lipid metabolism plays an important role in cancer. The aim of this study was to investigate the relationship between lipid metabolism and the development of cervical cancer, and to explore the prognostic significance of lipid metabolism-related genes in patients with cervical cancer.

**Methods:**

Initially, we retrospectively collected data from 1589 cervical cancer patients treated at the Affiliated Hospital of Qingdao University, with 1589 healthy individuals from the physical examination center serving as the control group. The correlation between their serum lipid levels and cervical cancer was analyzed. Subsequently, leveraging public databases, we conducted comprehensive studies on lipid metabolism-related genes. Additionally, we analyzed RNA expression profiling and clinical information sourced from TCGA and GTEx databases. Finally, we established a prognostic model integrating 9 genes associated with lipid metabolism and generated a nomogram model using R. GO and KEGG were performed to explore the functions and pathways of lipid metabolism-related genes.

**Results:**

Our findings revealed that patients with cervical cancer exhibited dyslipidemia, characterized by elevated levels of TC, TG, and LDL-C, alongside reduced HDL-C levels compared to controls (P<0.05). Interestingly, compared with early-stage patients, advanced patients had lower HDL-C level and higher LDL-C level. Regression analysis further highlighted high TC, TG, and LDL-C as significant risk factors for cervical cancer. Then a total of 188 lipid metabolism-related genes were identified and a prognostic signature based on 9 genes was established and validated. The results of the GO and KEGG functional analysis indicated that the lipid metabolism-related genes are primarily concentrated on pathways associated with fatty acid metabolism.

**Conclusion:**

Our study underscores the varying degrees of dyslipidemia observed in patients with cervical cancer, emphasizing the relevance of serum lipids in disease development. Our prognostic riskScore model predicted the overall survival time of patients based on 9 genes associated with lipid metabolism. These 9 genes may be tumor biomarkers and new targets for the treatment of cervical cancer.

## 1 Background

Cervical cancer (CC) stands as one of the most prevalent malignancies affecting the female reproductive system (1, 2). The Global Cancer Statistics Report stated that the incidence of CC ranks fourth among female malignant tumors worldwide, posing a serious threat to women’s health and a leading cause of death (3). This multifactorial disease involves intricate regulatory mechanisms in its occurrence and progression. Surgery, chemotherapy and radiotherapy are the main treatment methods at present (4). Recent years, with the development of screening and vaccination, the incidence of CC has been decreased worldwide. While in third world countries, the prevalence of CC has not been significantly decreased and is gradually showing a trend of youthfulness (5). Given this persistent challenge, it is of great clinical significance to find key targets and explore new diagnostic and therapeutic modalities for CC.

Lipid metabolism is fundamental in organisms, involving the absorption, synthesis, and breakdown of fats by various enzymes (6). Currently disorders of lipid metabolism have been shown to be associated with a variety of malignant tumors, such as liver cancer, lung cancer, endometrial cancer (7–9). On the one hand, researchers focus on the correlation between serum lipid levels and cancer due to difficulties in directly measuring lipid levels in tissues (10). It is worth noting that dyslipidemia varies across different forms of tumors. For instance, the levels of blood HDL-C and LDL-C were related to ER or PR positive breast cancer (11); the TG level was positively correlated with ovarian cancer risk in patients (12). However, in cervical cancer research, limited studies explore the relationship between serum lipids and the disease, leading to inconclusive findings. On the other hand, alterations in lipid metabolism gene expression play a crucial role in tumor behavior. Zhang et al. (13)confirmed that SQLE level was higher in CC tissues than those in paracancerous tissues and SQLE could promote CC progression by modulating the p53 signaling pathway. In addition, FABP4 has been demonstrated to be a risk factor for lymph node metastasis in patients with CC (14). Nevertheless, the prognostic value of lipid metabolism-related genes in cervical cancer remains uncertain, and systematic prognostic model construction for these biomarkers is lacking. In this study, we reviewed the relationship between serum lipid levels and the occurrence and development of CC. Then bioinformatics tools were used to explore LMRGs associated with CC prognosis. Finally, we established a prediction model for prognosis of CC aiming to provide guidance and support for the diagnosis and treatment of CC.

## 2 Methods

### 2.1 Patients and clinical information collection

This study adhered to the principles outlined in the Declaration of Helsinki and received approval from the Ethics Committee of the Affiliated Hospital of Qingdao University. Written informed consent was obtained from all participants. A total of 1589 patients with CC in the Affiliated Hospital of Qingdao University from January 2012 to December 2022 was retrospectively collected through the medical record system. The criteria for inclusion were as follows: (1) CC patients were diagnosed by definite histopathology; (2) patients with first diagnosed CC were not treated with surgery, chemotherapy or radiotherapy; (3) patients with complete clinical information. Pregnant women, Patients with other malignant tumors, diseases affecting blood lipid metabolism (hypertension, diabetes, familial hyperlipidemia, abnormal thyroid function, etc.), and taking drugs that affect blood lipid levels (hormones, lipid-lowering agent, etc.) were excluded. Patients with BMI<18.5 and BMI>28.0 (kg/m2) were also excluded due to the impact of obesity on blood lipid levels (15). Another 1589 healthy individuals were included during the same period as the controls through the physical examination center system (16). The exclusion criteria for controls were the same as those for CC patients.

Relevant clinical information collected included age, body mass index (BMI), stage, histopathological results, and serum lipid profile. The 2018 International Federation of Gynaecological and Obstetrics (FIGO) classification system was used for all CC patients (17). The blood samples were venous blood taken on an empty stomach before the initial treatment or the physical examination. The samples were detected by Beckman Coulter AU5800 biochemical analyzer (Beckman Coulter, USA) and the serum lipid levels were determined using kits. With reference to the clinical guidelines (18) and the Laboratory Department of the Affiliated Hospital of Qingdao University, the following lipid levels criteria were used in this study: TC: ≥ 5.20 mmol/L for the high level group; TG: ≥ 1.70 mmol/L for the high level group; HDL-C:<1.0 mmol/L for the low level group; LDL-C: ≥ 3.40 mmol/L for the high level group.

### 2.2 Public databases analysis

We downloaded RNA-seq data (FPKM format) for integration of gene expression data from the UCSC database (https://genome.ucsc.edu/) for integration of gene expression data from the TCGA (https://portal.gdc.cancer.gov/) and GTEx (https://www.gtexportal.org/) databases. Related clinical data of CC patients downloaded from the TCGA database. The GO database (http://geneontology.org/) and KEGG database (https://www.kegg.jp/) were used to characterize the functions of the genes. We validated the protein expression of 9 genes using the HPA database (https://www.proteinatlas.org/).

### 2.3 Prognostic risk model development and validation

The lipid metabolism genes set in this paper were derived from the article (DOI: 10.1155/2022/8227806) (**Supplementary File 1**), encompassing six distinct lipid metabolism pathways (**Supplementary File 2**). R software was used to integrate gene expression data and clinical data to obtain prognostic analysis datasets. Excluding patients with incomplete clinical information data and less than 30 days of overall survival (OS), 266 patients were included in this study. Then patients were divided 1:1 to training set or testing set at random. The screening, modeling and calculation of prognosis-related lipid metabolism genes were carried out in the training set. Meanwhile, the testing and total cohorts were used to verify the performance of the model. The formula for calculating risk scores of LMR-DEGs was described as: Risk Score (RS) = $\sum_{i=1}^{n}$ Coef × Exp_i_. *Coef* represents the regression coefficient, *Exp* represents the gene expression level, and *n* represents the number of genes. To investigate the impact of clinical and pathological factors on patients survival, we performed Univariate and Multivariable Cox regression analysis. Eventually, a nomogram was built to predict an individual’s probability of survival.

### 2.4 Statistical analysis

SPSS 25.0 was used for the analysis of clinical data. The quantitative data were described as mean ± standard deviation ( $\bar{x}$ ± s). *Student’s t test* was used to analyze the difference between two groups. *P* less than 0.05 was regarded as statistically significant. R (version 4.3.1) is a free statistical software, differential gene expression analysis, Cox regression analysis, LASSO analysis and nomogram model establishment were performed using it.

## 3 Results

### 3.1 Serum lipid levels between CC and control groups

Detailed information on the two groups were provided in **Supplementary File 3**. Age and BMI were not significantly different between the two groups (**Table 1**, *P*>0.05), which were comparable. Compared with the control group, the cancer group had higher TC, TG, LDL-C levels and lower HDL-C level (**Table 1**, *P*<0.05).

**Table 1:** Comparative analysis between CC group and control group.

Characters	CC group (n=1589)	Control group (n=1589)	t	*P*
Age(years)	48.77 ± 9.51	48.52 ± 7.39	-0.827	0.408
BMI(kg/m^2^)	23.64 ± 2.28	23.50 ± 2.08	1.868	0.062
TC (mmol/L)	4.88 ± 1.40	4.75 ± 0.72	3.179	0.001^*^
TG (mmol/L)	1.22 ± 0.96	0.99 ± 0.75	7.577	<0.001^*^
HDL-C (mmol/L)	1.52 ± 0.35	1.57 ± 0.36	-4.454	<0.001^*^
LDL-C (mmol/L)	2.91 ± 0.78	2.48 ± 0.52	18.185	<0.001^*^

^*^P<0.05.

### 3.2 Comparison of serum lipid levels among different subgroups of CC

The patients were stratified into various subgroups based on their FIGO stage, histological types, pathological types, and lymph node metastasis status, with the results presented in **Table 2**. We observed a significant difference in HDL-C levels between early-stage CC patients and those with intermediate or advanced stages, with higher levels detected in the former group. Conversely, LDL-C levels were found to be lower in early-stage patients compared to those with intermediate or advanced stages (*P* < 0.05). However, there were no statistical differences in serum lipid levels among patients with different histological types, patients with different pathological types, or between patients with or without lymph node metastasis (**Table 2**, *P>*0.05).

**Table 2:** Comparison of different clinical and pathological characteristics of serum lipid levels in patients with CC.

Characters(n)	TC (mmol/L)	TG (mmol/L)	HDL-C (mmol/L)	LDL-C (mmol/L)
Histological types				
G1+G2(1089)	4.88 ± 1.42	1.25 ± 1.06	1.52 ± 0.35	2.92 ± 0.76
G3(500)	4.88 ± 1.39	1.17 ± 0.70	1.51 ± 0.36	2.89 ± 0.81
*P*	0.972	0.105	0.544	0.464
FIGO stage				
I(951)	4.83 ± 1.43	1.20 ± 1.03	1.53 ± 0.35	2.86 ± 0.74
II-IV(638)	4.95 ± 1.36	1.25 ± 0.85	1.49 ± 0.36	2.98 ± 0.83
*P*	0.106	0.306	0.023^*^	0.005^*^
Pathological types				
Squamous cell carcinoma(1322)	4.90 ± 1.39	1.23 ± 1.00	1.51 ± 0.35	2.92 ± 0.78
Non-squamous cell carcinoma(267)	4.76 ± 1.47	1.18 ± 0.75	1.52 ± 0.36	2.84 ± 0.77
*P*	0.127	0.373	0.760	0.147
Lymphatic metastasis				
Negative(1371)	4.89 ± 1.39	1.24 ± 0.99	1.51 ± 0.35	2.92 ± 0.78
Positive(218)	4.77 ± 1.44	1.15 ± 0.76	1.53 ± 0.37	2.85 ± 0.78
*P*	0.228	0.213	0.480	0.278

^*^P<0.05.

### 3.3 Logistic regression analysis of lipid levels values of CC

The occurrence of CC served as the outcome variable, while serum lipid levels were utilized as variables for logistic regression analysis. Lipid levels were divided into normal and abnormal groups based on reference standards. Age and BMI were also included in the regression as continuous variables. The results indicated that TC, TG, LDL-C levels were positively correlated with CC and were risk factors affecting the occurrence of CC (**Table 3**). Age as a protective factor in the development of CC. In contrast, low level of HDL-C and BMI had no significant effect on CC carcinogenesis.

**Table 3:** Results of multivariate Logistic regression.

	β	SE	Wald	*P*	OR	95%CI
Age	-0.016	0.005	11.501	0.001	0.985	0.976-0.993
BMI	0.003	0.017	0.039	0.844	1.003	0.970-1.038
TC	0.177	0.086	4.247	0.039^*^	1.193	1.009-1.412
TG	0.571	0.116	24.301	<0.001^*^	1.771	1.411-2.222
HDL-C	0.275	0.163	2.850	0.091	1.316	0.957-1.811
LDL-C	1.964	0.152	166.167	<0.001^*^	7.129	5.288-9.609

^*^P<0.05.

### 3.4 Identification of LMR-DEGs in CC

**Figure 1:**
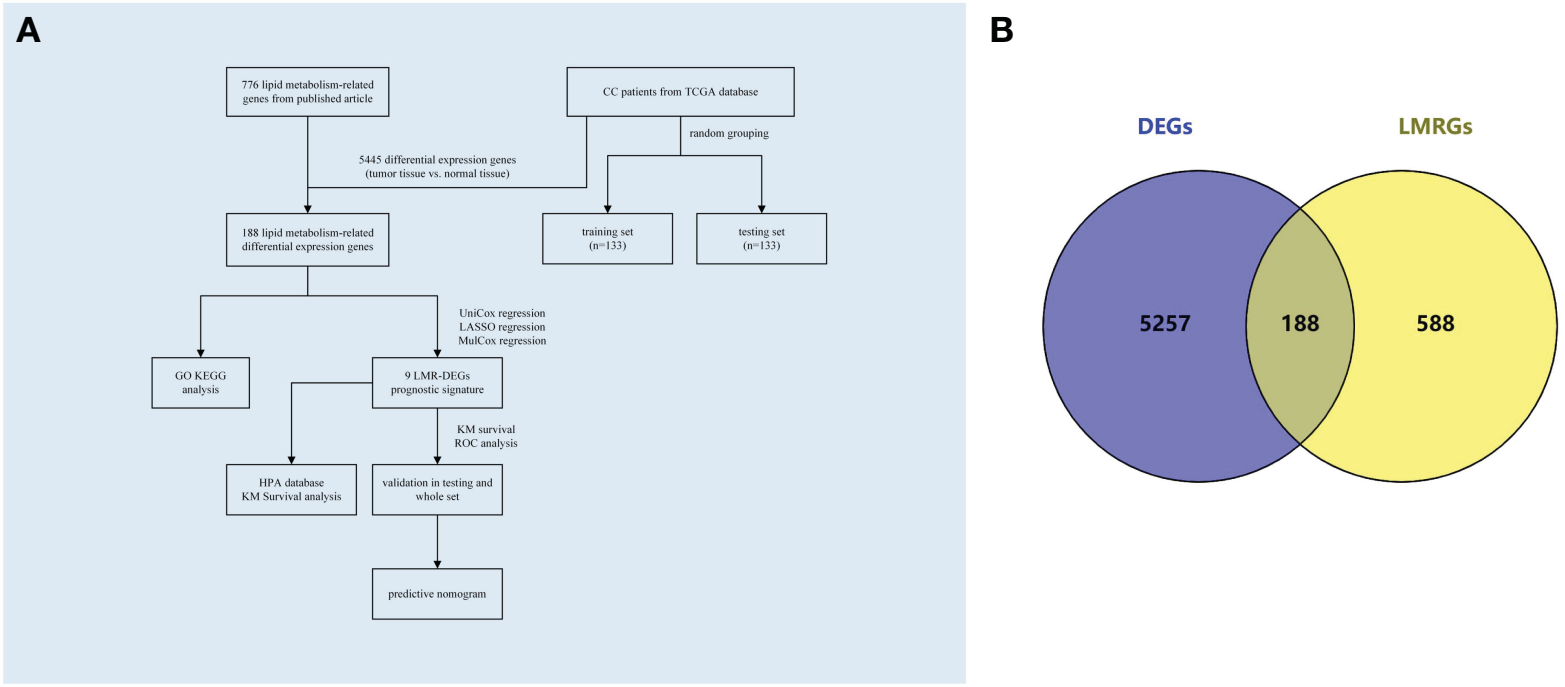
Flowchart and Venn diagrams. **(A)** Study flowchart **(B)** Blue represents the DEGs in CC; Yellow represents LMRGs; the intermediate intersection section is the LMR-DEGs.

The flowchart depicting the methodology of this study was illustrated in **Figure 1A**. Baseline clinical information downloaded from TCGA database of CC patients were shown in **Supplementary File 4**. Gene expression data and clinical information of all CC patients were screened from the TCGA database; gene expression data of normal cervical tissues were obtained by screening from the GTEx database. Differential analysis of the genes expression data using the “limma” package of R (4.3.1) software obtained 5445 differential genes including 2836 down-regulated and 2609 up-regulated genes (| log FC |> 1, P adj<0.05). Subsequently, a total of 776 lipid metabolism-related genes sourced from published literature were integrated into the analysis. By intersecting the differential genes with the lipid metabolism gene set, 188 differential genes associated with lipid metabolism were identified (**Figure 1B**).

### 3.5 Functional enrichment analysis

**Figure 2:**
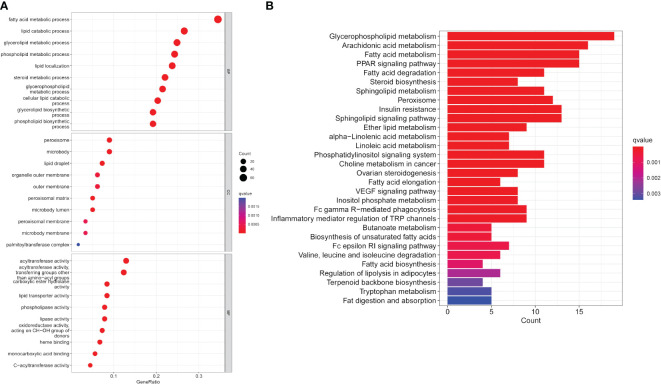
Functional enrichment analysis of expression profile data of patients. **(A)** GO annotation. **(B)** The KEGG pathway analysis.

GO enrichment analysis of LMRGs in CC revealed significant enrichment across molecular functions, biological processes, and cellular components. Specifically, LMRGs exhibited notable enrichment in fatty acid metabolism and lipid catabolism in biological processes. In terms of cellular components, LMRGs were primarily concentrated in peroxisomes and lipid droplets. Furthermore, molecular function analysis indicated enrichment in acyltransferase and carboxylate hydrolase activity (**Figure 2A**). Moreover, KEGG analysis suggested that these genes were mainly concentrated in glycerophosphate metabolic pathway, arachidonic acid metabolic pathway, fatty acid metabolic pathway and PPAR (peroxisome proliferator activated receptor) signal pathway (**Figure 2B**).

### 3.6 Construction of a LMR-DEGs prognostic risk model in CC

**Figure 3:**
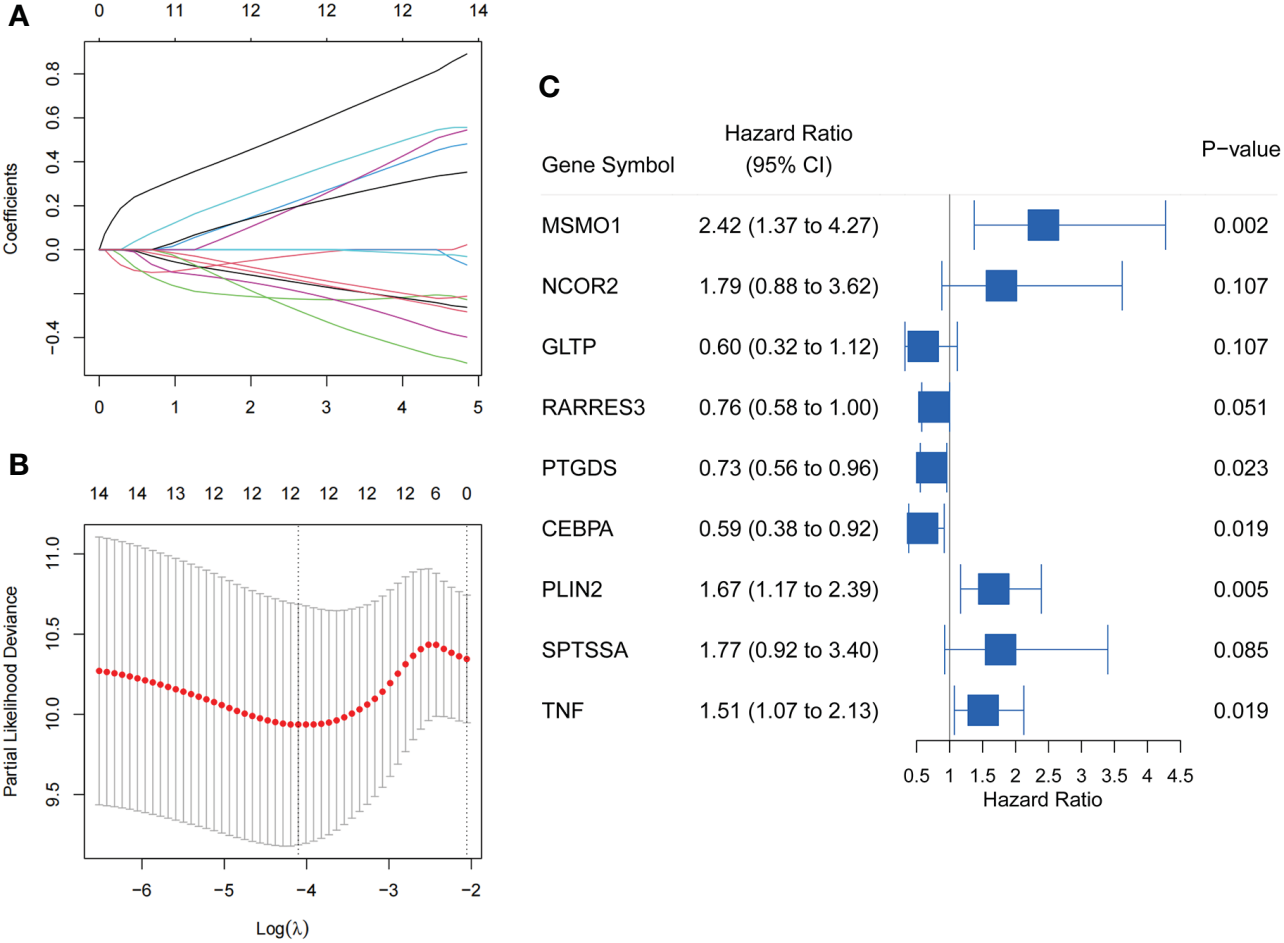
Screening of LMR-DEGs with prognostic significance in training set. **(A)** Trajectories of variable coefficients in LASSO regression. **(B)** LASSO regression cross-validation parameter selection process. **(C)** Forest plot of 9 LMR-DEGs by Multivariate Cox regression analysis.

In our study, 266 CC patients were divided 1:1 to training set or testing set at random. Then, we integrated the data on the expression of lipid metabolism and survival information of 133 patients in the training set. Through Univariable Cox regression analysis, we identified 14 genes significantly associated with OS (*P<*0.05) (**Supplementary File 5**). Following this, 9 LMR-DEGs with significant prognostic significance in CC were identified by LASSO regression (**Figures 3A, B**) and Multivariate Cox regression analysis for the construction. The prognostic riskScore formula: Risk Score (RS) = Exp (MSMO1) * 0.885 + Exp (NCOR2) * 0.580 – Exp (GLTP) * 0.510 – Exp (RARRES3) * 0.273 – Exp (PTGDS) * 0.316 – Exp (CEBPA) * 0.526 + Exp (PLIN2) * 0.513 + Exp (SPTSSA) * 0.573 + Exp (TNF) * 0.412. Forest plot of 9 genes was produced (**Figure 3C**).

### 3.7 Validation of a LMR-DEGs prognostic risk model in CC

**Figure 4:**
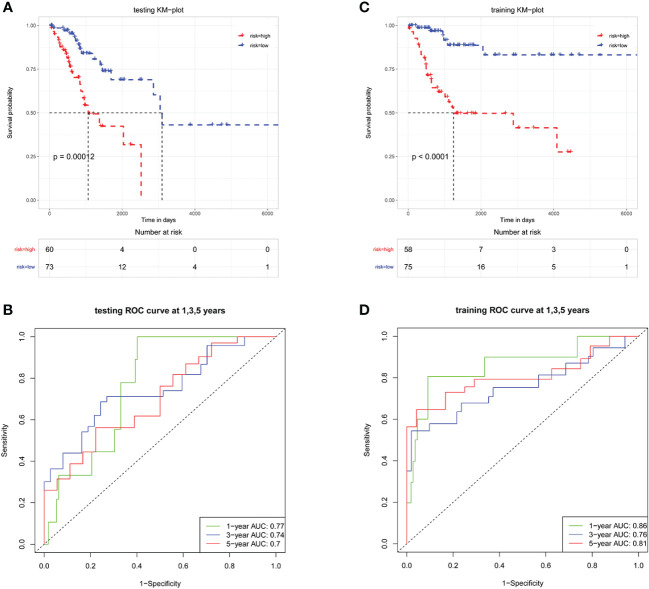
LMR-DEGs-based risk models have favorable predictive ability in the training and testing sets. Kaplan-Meier analysis in training set **(A)** and testing set **(C)**. ROC analysis in training set **(B)** and testing set **(D)**, AUCs were assessed for 1-, 3-, 5-year survival.

Patients in the training cohort were classified into high-risk and low-risk groups according to the median riskScore. The Kaplan-Meier analysis showed that patients in the low-risk group had a significantly higher OS than those in the high-risk group (**Figure 4A**, *P*<0.0001). Moreover, the area under the Receiver operating characteristic (ROC) (AUCs) revealed that this prognostic model has a high predictive value in training cohort, with AUCs values of 1, 3 and 5 years were 0.86, 0.76, and 0.81 (**Figure 4B**). Then the prognostic riskScore formula was applied to the testing cohort, showing similar results to the training cohort (**Figures 4C, D**).

**Figure 5:**
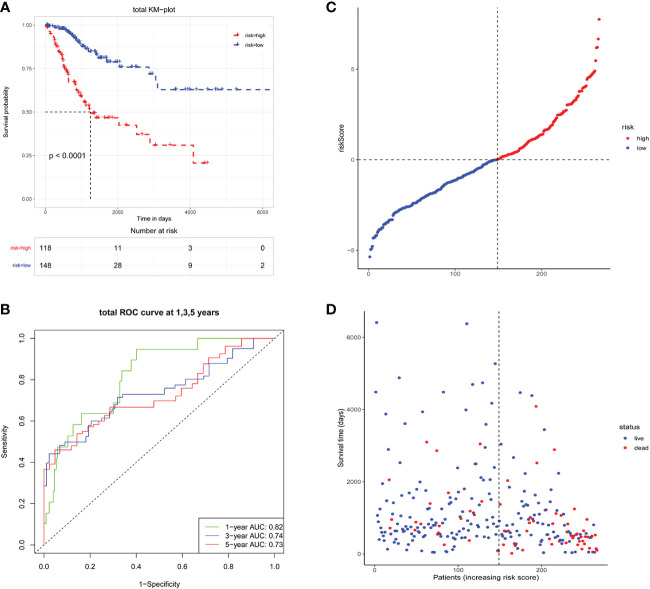
Survival analysis of total CC patients. **(A)** Kaplan-Meier analysis in total patients; **(B)** ROC curve analysis in total patients, AUCs were assessed for 1-, 3-, 5-year survival; **(C, D)** The description of risk scores, patient status and survival time.

Further validation showed that this model was applicable to all patients, with significantly different in OS between the high- and low-risk groups (**Figure 5A**). The ROC curves have also shown that the model had a good predictive ability for OS in patients with CC (**Figure 5B**). The distribution of risk scores and survival status was shown in **Figures 5C, D**. It is worth noting that when the riskScore increased, there were significantly more deaths in the high-risk group than in the low-risk group.

### 3.8 Development and evaluation of a LMR-DEGs clinicopathologic nomogram in CC

**Figure 6:**
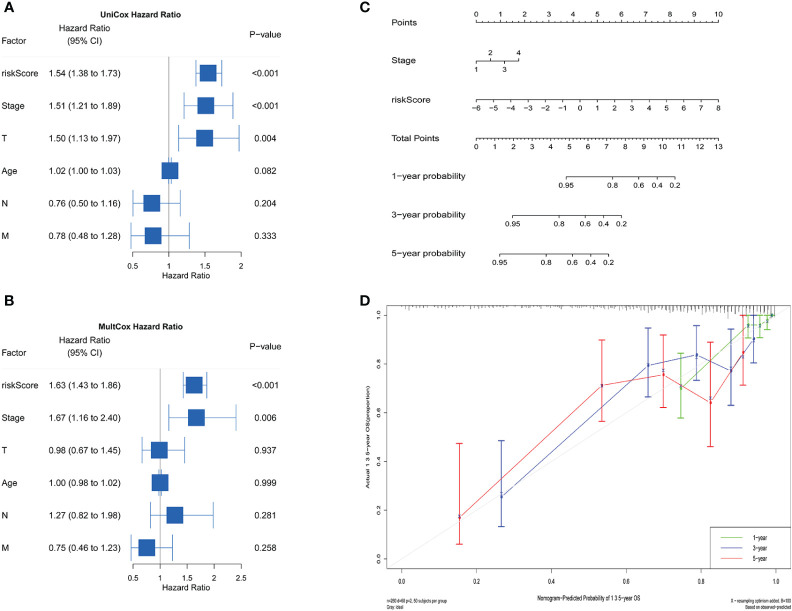
Independent prognostic analysis and construction of the nomogram. Univariate **(A)** and Multivariate **(B)** Cox analysis suggested that the riskScore and FIGO stage were independent risk factors for predicting prognosis. **(C)** A nomogram for estimating the prognosis of patients with CC. **(D)** Correction chart for 1-, 3-, 5-year predicted survival.

Univariate and Multivariate Cox regression analyses combined with clinical data were conducted to evaluate the impact of riskScore and clinical parameters on patients’ prognosis (**Figures 6A, B**). Our findings indicated that riskScore (HR=1.54, *P*<0.001), T-staging (HR=1.50, *P=*0.004) and FIGO stage (HR=1.51, *P*<0.001) were all significant factors influencing the prognosis of patients with CC in the Univariate Cox analysis. Further Multifactorial regression analysis showed that riskScore (HR=1.63, P< 0.001) and FIGO stage (HR=1.67, P=0.006) could be considered as an independent predictor of survival in CC patients. Moreover, we constructed a nomogram for individual survival predictions in CC patients, incorporating riskScore and FIGO stage (**Figure 6C**). This risk model could be based on the actual situation of patients to find the corresponding score and the cumulative score enabled the survival rate of the patients at 1, 3, and 5 years to be calculated. The quality of the model was further verified by plotting calibration curves, which showed that the survival rate of patients were similar to the actual OS (**Figure 6D**).

### 3.9 Expression and prognostic analysis of genes in risk model

**Figure 7:**
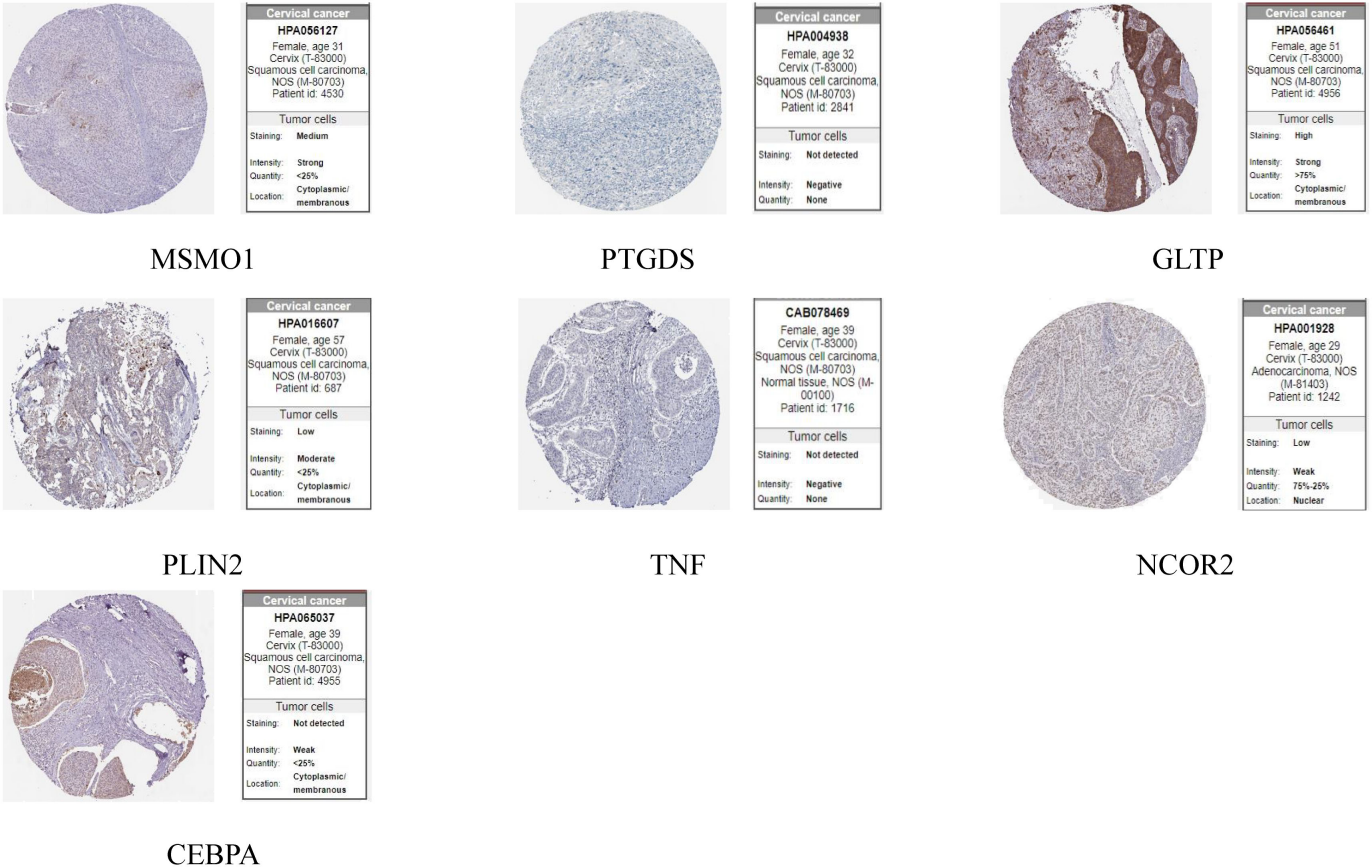
Expression of 9 LMR-DEGs at the protein level.

**Figure 8:**
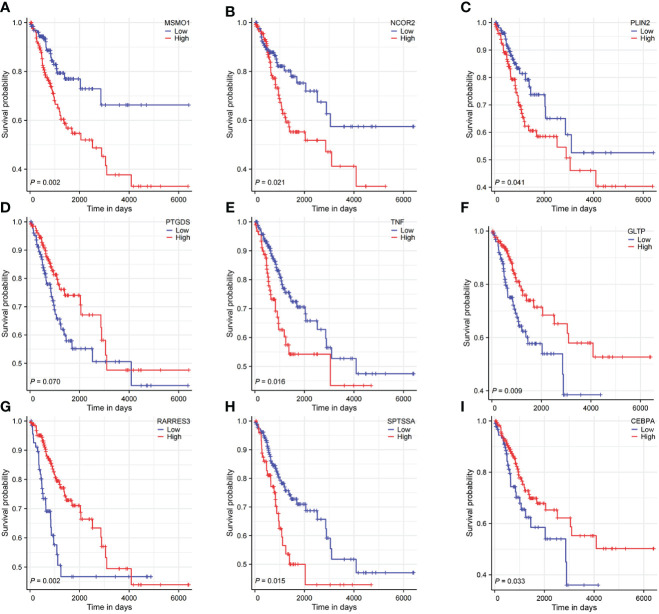
Survival analysis of 9 LMR-DEGs. **(A-I)** representing MSMO1, NCOR2, PLIN2, PTGDS, TNF, GLTP, RARRES3, SPTSSA, CEBPA.

**Figure 9:**
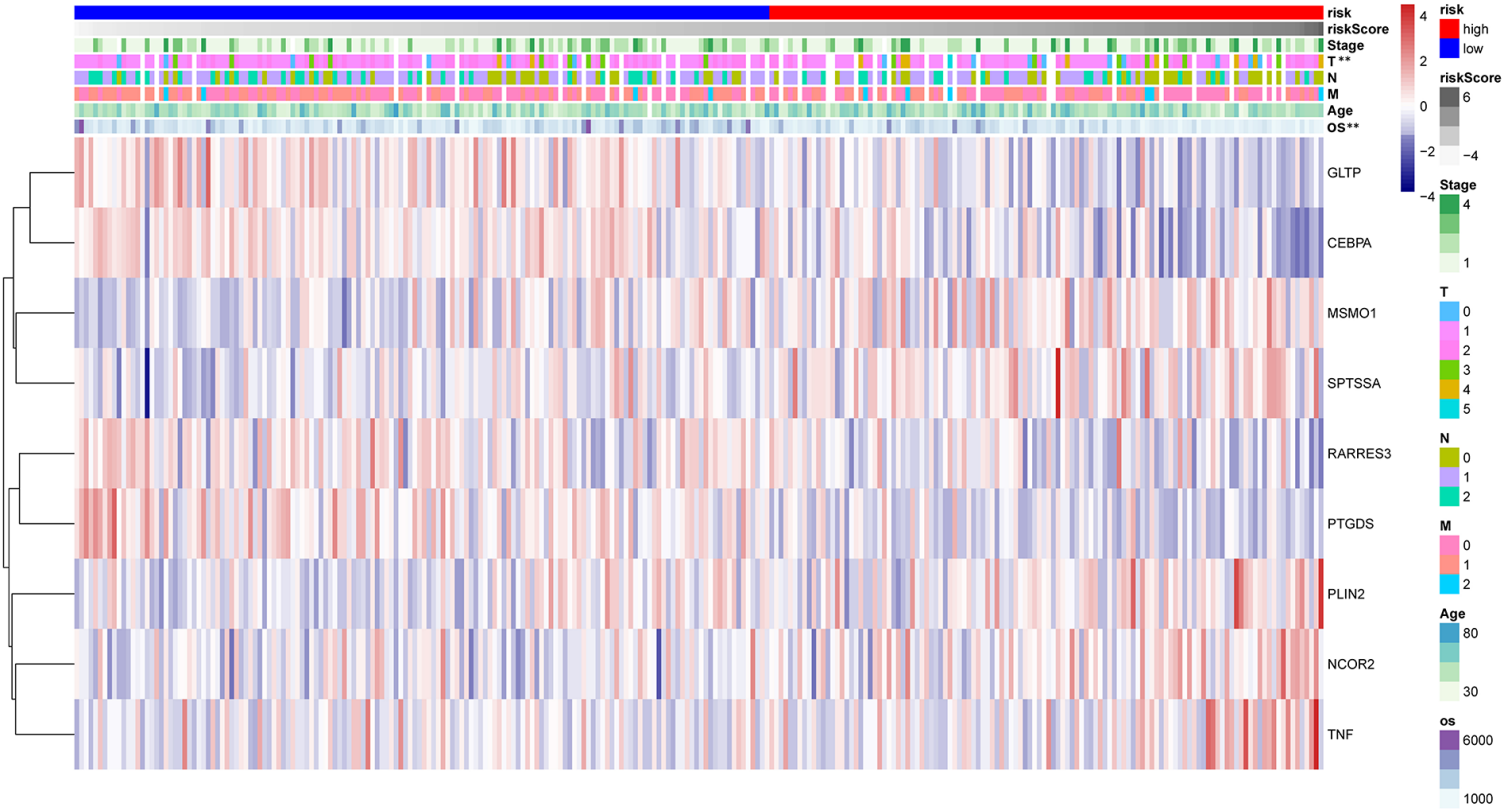
The heatmap reflecting 9 LMR-DEGs expression and clinicopathologic characters associated with CC in high- and low-risk CC patients. **P<0.01.

**Figure 10:**
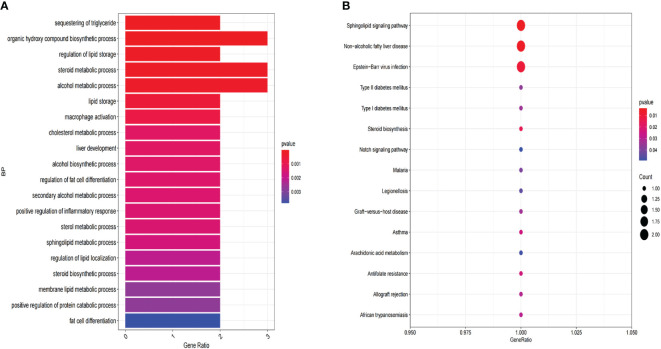
Functional enrichment analysis of 9 LMR-DEGs. **(A)** GO annotation. **(B)** The KEGG pathway analysis.

The HPA database was utilized for immunohistochemical analysis of genes, although SPTSSA and RARRES3 were not available in the database. As depicted in **Figure 7**, PTGDS and TNF were not found to be significantly expressed in CC cells. The expression trends of the remaining genes were consistent with our findings. The Kaplan–Meier survival curves also confirmed that higher expression of MSMO1, NCOR2, PLIN2, SPTSSA and TNF and lower expression of GLTP, RARRES3, PTGDS and CEBPA were associated with worse OS in the TCGA dataset (**Figures 8A–I**). We constructed a heatmap containing gene expression patterns and clinicopathological factors (**Figure 9**). Once again, the gene expression pattern corroborated our earlier analyses. We did not find statistically significant differences between riskScore and N stage, M stage, FIGO Stage and age. However, we noted that higher riskScore were associated with a higher T stage and poorer OS. Functional analysis of these 9 genes was performed using the GO and KEGG databases. The results indicated that these genes are enriched in pathways related to lipid metabolism (**Figure 10**).

## 4 Discussion

Tumor cells exploit alterations in energy metabolism to drive their rapid growth and proliferation, a hallmark of malignancy (19). As an integral part of cells, lipids play an crucial role in maintaining cells survival as energy sources, signaling molecules, and second messengers (20). Growing evidence underscores the significant involvement of lipid metabolism in tumor progression (21). In terms of lipid metabolism, the most closely related to clinical practice is serum lipid levels, and the role of dyslipidemia in various cancers has been confirmed. For instance, high TC level could increase the risk of breast cancer recurrence (22). Similarly, studies in Korean populations have linked lower HDL-C and higher LDL-C levels with gastric cancer risk (23). Higher TC and LDL-C were significantly correlated with an increased risk of aggressive prostate cancer (24). Therefore, our study selected four main blood lipid indicators: TC, TG, HDL-C and LDL-C for research.

Our results showed that these four lipid parameters were significantly different between the two groups. TC, TG, LDL-C levels were differently increased in the CC group, while the HDL-C was decreased. Subsequently, the results of Logistic regression analysis indicated that high TG, TC, and LDL-C levels could increase the risk of CC. Interestingly, our results suggested that age was a protective factor in this study. This may be due to the increasing incidence of CC among young individuals in recent years and the relatively young age of our study population. However, our findings contradict some previous studies on serum lipid levels and CC. Sun et al. (25) did not observe a significant difference in blood lipid levels between patients and controls, which could be attributed to differences in sample size. Another study suggested that the occurrence of CC was associated with low TC level, which was contrary to our results (26). We hypothesized that this discrepancy may stem from variations in regulatory mechanisms affecting TC levels. On the one hand, cholesterol is consumed as a nutrient during the proliferation of cancer cells, so its level decreases with the progression of the disease. On the other hand, high cholesterol level induces abnormal pathological processes that cause cellular carcinogenesis, leading to a high cholesterol predisposition in cancer patients (27). As another transport form of TC, the trend of LDL-C is usually in line with that of TC. This was also demonstrated by our results, and the changes in LDL-C were more significant than in TC. Consistent with our results, most studies have concluded that high TG level was a risk factor for cancer development. The findings of Ulmer et al. (28)showed that high TG level was positively associated with the risk of CC. This may be due to high TG level activating the fatty acid pathway to generate reactive oxygen species (ROS) and inducing oxidative stress processes, which ultimately cause cellular damage contributing to carcinogenesis (29). The relationship between lipid metabolism (especially serum lipid levels) and tumor progression has been less well studied. Consequently, we examined the correlation between lipid levels and different clinical subgroups. The results revealed that low HDL-C level and high LDL-C level were associated with later staging of CC patients. Similarly, Zhang Wei et al. (30)found that low HDL-C level can increase the risk of cervical disease, while elevated LDL-C level was associated with progression of cervical lesions. HDL-C has been demonstrated to be anti-oxidant and anti-inflammatory in several studies (31), so we believed that HDL-C may inhibit the process of cancer development by preventing the oxidation of certain substances or reducing the aggregation of pro-inflammatory factors. In addition, our study did not find a significant association between lipid levels and histological type, pathological type and lymph node metastasis. Since our study was a single-center study, whether lipid levels correlate with clinical subgroups of CC requires further study.

Our study has established an association between lipid metabolism and CC, prompting us to delve into the significance and prognostic value of LMRGs in CC. Our goal was to devise an effective method for exploring lipid metabolism in CC patients and predicting clinical outcomes. Molecular prognostic markers can dynamically respond to tumor progression, so integrating multiple molecular markers to create a genetic model has better predictive efficacy than a single marker. Such an approach holds considerable promise for refining clinical decision-making processes. In this study, we developed a prognostic risk score model based on nine genes, employing a suite of bioinformatics techniques. Based on Univariate and Multivariate Cox regression we found that riskScore and FIGO stage were independent predictors of poor prognosis in CC patients. Meanwhile, we found that the LMR-DEGs in CC were mainly concentrated in fatty acid metabolism and lipolytic metabolism. The aberrant expression of these genes facilitates to satisfy the characteristics of large energy consumption of tumor cells and ensures their rapid growth. Moreover, to validate our findings, we cross-referenced protein expression levels using the HPA database.

Three genes in this model have been previously reported to be associated with the development of CC. MSMO1 is one of the key enzymes in the cholesterol synthesis pathway, and its expression in CC has been associated with the immune microenvironment and poor prognosis. Similarly, its high expression has been also shown to be associated with malignant behavior in other cancer, such as pancreatic cancer. PTGDS, a member of the lipid transport protein family, is associated with various pathways, including the MAPK signaling pathway. However, its precise effects in several solid tumors remain uncertain. Jiang et al. found that PTGDS is lowly expressed in CC and predict poor prognosis, which is consistent with our findings. Sphingolipid metabolism constitutes a vital branch of lipid metabolism, wherein GLTP plays a crucial role in regulating the transport of sphingolipids. Consistent with the results we obtained, the study by Shi et al. also found that GLTP was highly expressed in CC. Interestingly, patients with high expression have a better prognosis. This may be related to the different roles played by GLTP in the occurrence and development of cancer. SPTSSA is its newly discovered key enzyme, for which there are relatively few reports. SPTSSA was highly expressed in gliomas and significantly correlated with infiltrating immune cells and overall survival. Similar to these tumors, our study found that it was highly expressed in CC.

Lipid droplets are lipid-storing organelles with critical functions in energy homeostasis and cancer aggressiveness. PLIN2 is a marker of lipid droplets whose role in cancer has been controversial. In hepatocellular carcinoma, PLIN2 shows a trend of high expression and correlates with poor prognosis; however, in uterine smooth muscle tumors, low expression of PLIN2 reprograms the cells to a hyperproliferative phenotype. In the present study, we found that PLIN2 is lowly expressed in CC and may be associated with better prognosis. In summary, the specific role of PLIN2 in CC still needs further research. RARRES3, as a tumor suppressor, can act on various signaling pathways such as MAPK and PI3K pathway. It exhibited a high expression trend in CC and was related to better survival. However, in colon or breast cancer, its low expression is significantly associated with lymph node metastasis. It is well known that TNF is closely associated with cancer-related inflammation and malignant biological behavior. It is highly expressed in most malignant tumors, including CC in our exploration. The transcriptional co-repressor protein NCOR2, it has been proven to be associated with various metabolic related cancers such as breast cancer and prostate cancer. In our report, we found that NCOR2 was low expressed in CC. However, prognostic analysis showed that high-risk populations have poorer prognosis. This may be related to the transcriptional silencing of certain target genes mediated by this gene and its involvement in multiple complex pathways. CEBPA is a transcription factor that regulates the differentiation of adipocytes. In our early research, its expression can affect the prognosis of CC patients.

While our research has yielded significant insights into the relationship between lipid metabolism and CC, it is imperative to acknowledge several limitations that warrant consideration. Firstly, the retrospective nature of our study introduces inherent limitations, including the potential for selection bias. Future prospective studies could provide valuable insights by mitigating this bias and offering a more comprehensive understanding of lipid metabolism in the context of CC. Secondly, the absence of longitudinal follow-up in clinical studies on lipid levels restricts our ability to elucidate the prognostic significance of lipid parameters in CC patients. Long-term follow-up studies are essential to discern the dynamic interplay between lipid metabolism and disease progression. Lastly, while our prognostic model demonstrates promising predictive performance, its validation is confined to internal datasets. External validation in independent cohorts is essential to ascertain the generalizability and robustness of our model, thereby enhancing its clinical utility and facilitating broader adoption in CC management. Addressing these limitations will be pivotal in advancing our understanding of lipid metabolism in CC and translating these findings into meaningful clinical applications.

In conclusion, the study comprehensively analyzed the relationship between lipid levels and CC, providing data support for further research. Through the integration of gene expression and clinical data from CC patients, we have developed a risk model related to lipid metabolism. This model clearly illustrated the significance of lipid metabolism in the prognosis of CC. In the future, we should be more attention to the abnormalities of lipid levels and LMRGs in CC to provide new strategies for the clinical diagnosis and therapy of CC.

## 5 Conclusion

There was a correlation between serum lipids and the occurrence and development of CC. TC, TG, LDL-C may be risk factors for CC. Our study identified 9 LMRGs signatures associated with prognosis, and the predictive model may be an independent prognostic factor for CC.

## Data availability statement

The datasets presented in this study can be found in online repositories. The names of the repository/repositories and accession number(s) can be found in the article/**Supplementary Material**.

## Ethics statement

The studies involving humans were approved by The Medical Ethics Committee of Affiliated Hospital of Qingdao University. The studies were conducted in accordance with the local legislation and institutional requirements. The human samples used in this study were acquired from primarily isolated as part of your previous study for which ethical approval was obtained. Written informed consent for participation was not required from the participants or the participants’ legal guardians/next of kin in accordance with the national legislation and institutional requirements. Written informed consent was obtained from the individual(s) for the publication of any potentially identifiable images or data included in this article.

## Author contributions

LC: Writing – original draft, Writing – review & editing. ZL: Data curation, Writing – review & editing. QZ: Writing – review & editing. QY: Writing – review & editing.
