# Supplementary material for: Correlation study of serum lipid levels and lipid metabolism-related genes in cervical cancer
Source: Front Oncol. 2024 May 8;14:1384778. doi: 10.3389/fonc.2024.1384778 (PMC11109420; doi:10.3389/fonc.2024.1384778)
Supplement: Supplementary file 2 [file Table_2.doc]

**Supplementary file 2 : Six pathways involved in lipid metabolism.**

| **Pathway Database** **Gene Count** |
| --- |
| Peroxisome proliferator activated receptor alpha Reactome 119  Metabolism of lipids Reactome 738  Transcriptional regulation of white adipocyte differentiation Reactome 84  Sphingolipid metabolism Reactome 89  Glycerophospholipid metabolism KEGG 77  Fatty acid metabolism Reactome 177 |
| Total: 1284  Unique: 776 |
