# Supplementary material for: Correlation study of serum lipid levels and lipid metabolism-related genes in cervical cancer
Source: Front Oncol. 2024 May 8;14:1384778. doi: 10.3389/fonc.2024.1384778 (PMC11109420; doi:10.3389/fonc.2024.1384778)
Supplement: Supplementary file 3 [file Table_3.docx]

**Supplementary file 3**

**Baseline data between cancer and control group**

| Characters | Cancer Group | | Control Group | |
| --- | --- | --- | --- | --- |
|  | Number | Proportion(%) | Number | Proportion(%) |
| Age(years) |  |  |  |  |
| Median | 49.0 | | 48.0 | |
| ≤50 | 905 | 60.0 | 1008 | 63.4 |
| >50 | 684 | 40.0 | 581 | 36.6 |
| BMI(kg/m^2^) |  |  |  |  |
| <24 | 867 | 54.6 | 893 | 56.2 |
| ≥24 | 722 | 45.4 | 696 | 43.8 |

| Clinical factors | Subgroup | Number |
| --- | --- | --- |
| Age(years) | ≤50 | 905 |
|  | >50 | 684 |
| FIGO stage | I | 951 |
|  | II | 333 |
|  | III | 267 |
|  | IV | 38 |
| histological types | G1 | 279 |
|  | G2 | 810 |
|  | G3 | 500 |
| pathological types | Squamous cell carcinoma | 1322 |
|  | Adenocarcinoma | 233 |
|  | Adenosquamous carcinoma | 24 |
|  | others | 10 |
| lymph node metastasis | negative | 1371 |
|  | positive | 218 |

**Baseline data of CC patients in different subgroups**
