# Supplementary material for: Correlation study of serum lipid levels and lipid metabolism-related genes in cervical cancer
Source: Front Oncol. 2024 May 8;14:1384778. doi: 10.3389/fonc.2024.1384778 (PMC11109420; doi:10.3389/fonc.2024.1384778)
Supplement: Supplementary file 4 [file Table_4.docx]

**Supplementary file 4**

Clinical information of CC patients from TCGA database

| **Characters** | **Number** |
| --- | --- |
| Age |  |
| ≤50 | 160 |
| >50 | 106 |
| FIGO Stage |  |
| I-II | 204 |
| III-IV | 56 |
| Unknown | 6 |
| T |  |
| Tis | 1 |
| T1-2 | 190 |
| T3-4 | 26 |
| Tx | 15 |
| Unknown | 34 |
| N |  |
| N0 | 116 |
| N1 | 52 |
| Nx | 64 |
| Unknown | 34 |
| M |  |
| M0 | 101 |
| M1 | 10 |
| Mx | 39 |
| Unknown | 36 |
| Pathological types |  |
| Squamous cell carcinoma | 219 |
| Adenocarcinoma | 47 |
| Treatment type |  |
| Radiation Therapy | 128 |
| Pharmaceutical Therapy | 138 |
